# Glucose and Amino Acid Metabolic Dependencies Linked to Stemness and Metastasis in Different Aggressive Cancer Types

**DOI:** 10.3389/fphar.2021.723798

**Published:** 2021-09-13

**Authors:** Andrea Chisari, Irene Golán, Sabrina Campisano, Caroline Gélabert, Aristidis Moustakas, Patricia Sancho, Laia Caja

**Affiliations:** ^1^ Department of Chemistry, School of Sciences, National University of Mar del Plata, Mar del Plata, Argentina; ^2^ Department of Medical Biochemistry and Microbiology, Science for Life Laboratory, Biomedical Center, Uppsala University, Uppsala, Sweden; ^3^ Translational Research Unit, Hospital Universitario Miguel Servet, IIS Aragon, Zaragoza, Spain

**Keywords:** glucose, amino acid, PDAC-pancreatic ductal adenocarcinoma, GBM-glioblastoma multiforme, HCC-hepatocellular carcinoma, cancer stem cell (CSC), therapy

## Abstract

Malignant cells are commonly characterised by being capable of invading tissue, growing self-sufficiently and uncontrollably, being insensitive to apoptosis induction and controlling their environment, for example inducing angiogenesis. Amongst them, a subpopulation of cancer cells, called cancer stem cells (CSCs) shows sustained replicative potential, tumor-initiating properties and chemoresistance. These characteristics make CSCs responsible for therapy resistance, tumor relapse and growth in distant organs, causing metastatic dissemination. For these reasons, eliminating CSCs is necessary in order to achieve long-term survival of cancer patients. New insights in cancer metabolism have revealed that cellular metabolism in tumors is highly heterogeneous and that CSCs show specific metabolic traits supporting their unique functionality. Indeed, CSCs adapt differently to the deprivation of specific nutrients that represent potentially targetable vulnerabilities. This review focuses on three of the most aggressive tumor types: pancreatic ductal adenocarcinoma (PDAC), hepatocellular carcinoma (HCC) and glioblastoma (GBM). The aim is to prove whether CSCs from different tumour types share common metabolic requirements and responses to nutrient starvation, by outlining the diverse roles of glucose and amino acids within tumour cells and in the tumour microenvironment, as well as the consequences of their deprivation. Beyond their role in biosynthesis, they serve as energy sources and help maintain redox balance. In addition, glucose and amino acid derivatives contribute to immune responses linked to tumourigenesis and metastasis. Furthermore, potential metabolic liabilities are identified and discussed as targets for therapeutic intervention.

## Introduction

### Cancer and Cancer Stem Cells

Cancer is one of the leading causes of death in modern society. Fourteen million new cancer cases are diagnosed and eight million people die of cancer yearly worldwide (Torre et al., 2016). Cancer is characterised by specific genetic alterations, genome-widespread epigenetic alterations and chromosomal aberrations. This leads to key genes either gaining or losing their molecular function and signaling pathways, and thus reflecting changes in the physiological function of the affected cell type and tissue. Typically, tumour cells develop from normal cells that make mistakes in their DNA repair mechanisms and accumulate mutations, which results in the acquisition of new properties, such as an uncontrolled cell division that usually leads to non-malignant tissue hyperplasia. Malignant cells are capable of de-differentiation, tissue invasion, nutrient recruitment through blood by angiogenesis induction, self-sufficient growth, insensitivity to negative growth control and apoptosis initiation, and limitless replicative potential (Hanahan and Weinberg, 2011).

The development of modern treatments and their application in specific cancer patients in combination with surgery (physical resection of the tumour), chemotherapy (use of drug combinations) or radiotherapy (high-energy radiation that damages DNA and kills cells), have led to a significant improvement in their overall survival rate. Unfortunately, the cure for cancer remains challenging due to the frequent relapse in patients that receive one or several of these modern treatments. This is due to cancer cells heterogeneity within each tumour, some of which develop resistance mechanisms to the treatment and manage to survive (Dagogo-Jack and Shaw, 2018). The usually small pool of resistant and surviving cells initiate and generate new tumours in the primary site, even after their temporary elimination, and progress by causing distant metastasis in secondary sites in the human body (Lawson et al., 2015). The cells that can initiate the new tumour outgrowth, which also exhibit treatment resistance, are often described as “tumour-initiating cells” or cancer stem cells (CSCs) (Diehn et al., 2009; Wang et al., 2010).

CSCs share some properties with normal adult stem cells, such as: 1) specific gene expression (Ginestier et al., 2007), 2) division by generating a CSC (self-renewal) and another cell that can later differentiate to diverse malignant cell types in a given tissue, and 3) the activation of common signalling pathways that contribute to proliferation, self-renewal and survival/resistance (Kanwar et al., 2010; Rossi et al., 2020). CSCs, however, differ from normal adult stem cells in that they remain fundamentally cancer cells and that the regulating mechanisms in normal stem cells are deregulated in CSCs. This leads to the continuous expansion and production of an aberrantly differentiated progeny: the tumour (Rossi et al., 2020). To complicate matters, CSCs are also thought to be generated by pre-malignant or malignant cells via a process of de-differentiation that makes tumour cells resemble embryonic cells to some extent. Therefore CSCs and embryonic cells may share common cell biological and molecular properties (Lambert and Weinberg, 2021). The understanding of CSCs is consequently essential to identify molecules that can be targeted for drug development, and thus improve the outcome of cancer treatments.

CSCs are also important for the metastatic dissemination of primary tumours to distant organs (Diehn et al., 2009; Wang et al., 2010). Whether CSCs generate cells with enhanced ability to invade and initiate metastasis as they proliferate, or their contribution to metastasis primarily involves growth after colonising new organs or tissues remains relatively unexplored. (Karuna Ganesh, 2013). Based on the latter, once circulating tumour cells manage to form micrometastases, they generate CSCs required to fuel the secondary tumour expansion to the metastatic site (Lambert and Weinberg, 2021). These important alternative function modes linking CSCs to metastasis are actively studied (Ganesh and Massagué, 2021).

This review focuses on three of the most aggressive tumour types, namely pancreatic ductal adenocarcinoma (PDAC), hepatocellular carcinoma (HCC) and glioblastoma (GBM). The aim is to understand and identify common CSCs metabolic requirements and responses to nutrient starvation, as well as potential metabolic vulnerabilities that can be exploited therapeutically.

### Brief Overview of Cancer Cell Metabolism

#### Glucose Metabolism

Cancer cells in solid tumours are subjected to high energy demands to support their accelerated growth rate. At the same time, essential nutrient supply, such as glucose, is affected by a dysregulated vasculature that results in a microenvironment characterised by low glucose, hypoxia and acidic pH (Wang X. et al., 2019). Thus, glucose deprivation and lactic acidosis are common adverse microenvironments in solid tumours (Duan et al., 2018), both consequence of an elevated glycolytic activity in tumour cells (Warbug effect). Besides producing energy, glucose metabolism supports the synthesis of metabolic building blocks, such as nucleotides and lipids, it provides NADPH to maintain the cellular redox state and, additionally, glucose is used for the post-translational modification (O-GlcNAcylation) of intracellular proteins that regulate nutrient sensing and stress response via the hexosamine biosynthetic pathway (HBP) (Lam et al., 2021). Due to the essential role of glucose in cancer cells proliferation and survival, transporters and enzymes involved in glucose metabolism are frequently up-regulated in cancer cells (Yamaguchi et al., 2020). Glycolysis was initially postulated as the main metabolic feature in cancer cells. However, it is currently accepted that most cancers still produce ATP via mitochondrial oxidative phosphorylation (OXPHOS) and modulate the contribution of glycolytic and OXPHOS pathways in different phases of the cell cycle or in response to environmental factors, such as oxygen availability (De Berardinis and Chandel, 2016). For example, cells located close to blood vessels may favour OXPHOS due to the relatively higher oxygen concentration (Talasila et al., 2017), whereas cells closer to the hypoxic tumour areas favour glycolysis.

#### Amino Acid Metabolism

**FIGURE 1:**
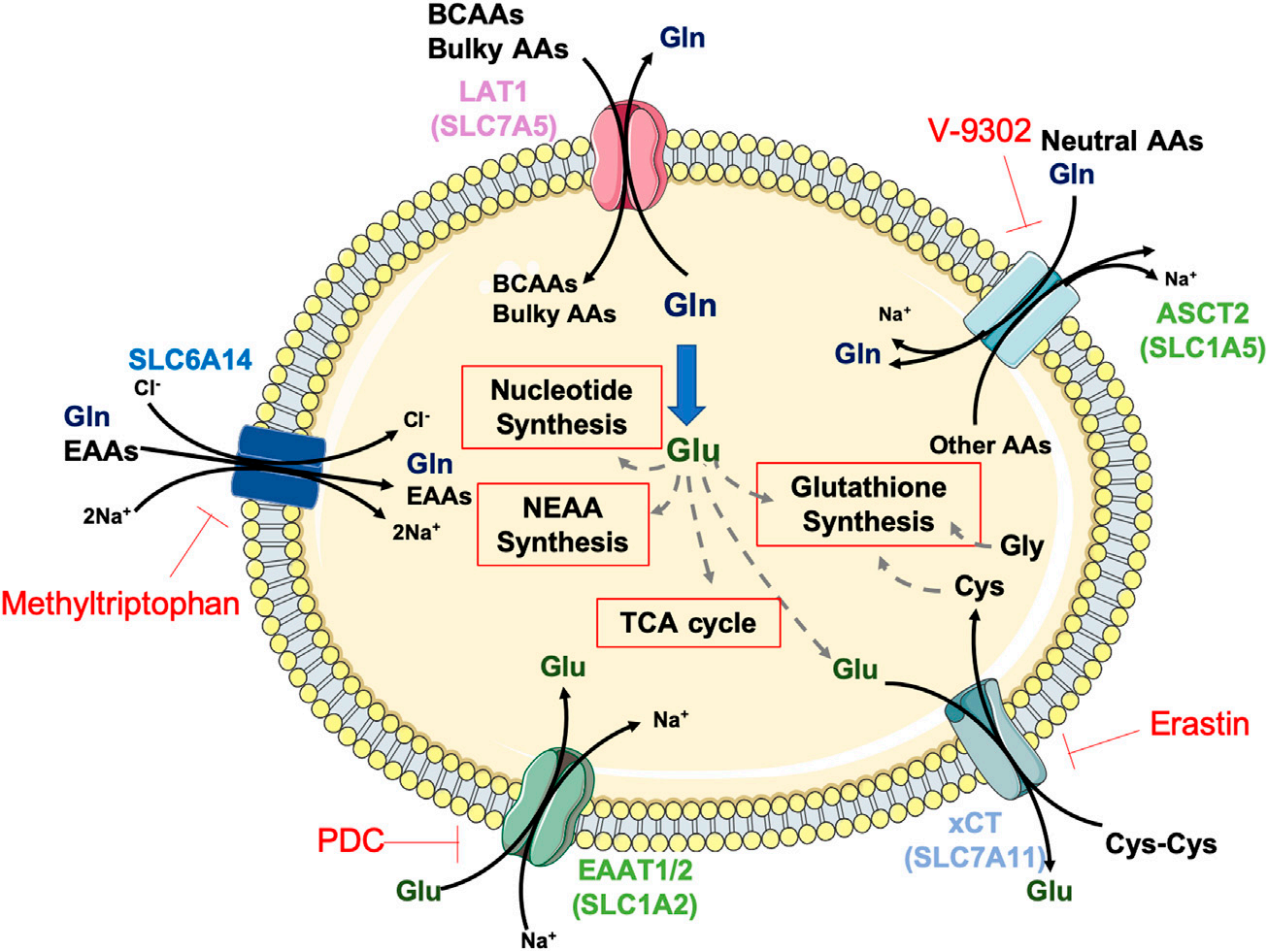
Amino acid transporters and metabolism in aggressive cancers. The amino acids (AAs) glutamate (Glu, dark green) and glutamine (Gln, dark blue) are key players for amino acid transport and global cellular metabolism in PDAC, HCC and GBM tumours. Glutamine transported into the tumour cells by the transporters SLC6A14 or ASCT2 is metabolised to glutamate via glutaminolysis or serves as substrate for the antiport of branched-chain (BCAAs) and bulky amino acids via LAT1. Glutamate facilitates cysteine (Cys) uptake via xCT or supports key energy and biosynthetic pathways, such as glutathione, non-essential AA and nucleotides synthesis or TCA cycle anaplerosis. Depicted in red, AA transporters inhibitors.

Mammals cannot synthesise all the necessary amino acids for protein synthesis and must acquire nine out of twenty amino acids from the diet, called essential amino acids (EAA: histidine, isoleucine, leucine, lysine, methionine, phenylalanine, threonine, tryptophan and valine). Although, by definition, all the non-essential amino acids (NEAAs: alanine, arginine, asparagine, aspartic acid, cysteine, glutamine, glutamic acid, glycine, proline, serine, tyrosine) can be synthesised *de novo*, recent evidence indicates that some cancer cells may show specific amino acid requirements, becoming dependent on conditionally essential amino acids (CEAAs). For that reason, metabolic reprogramming in cancer is also characterised by enhanced amino acid uptake and *de novo* metabolism of otherwise NEAAs (Fuchs and Bode, 2005; Dunphy et al., 2018). Indeed, cancer cells upregulate amino acid transporters to transfer them across the plasma membrane. Amongst the transporters, SLC6A14 shows the broadest substrate selectivity encompassing all EAAs and glutamine (Figure 1), and it is upregulated in several cancers (Lieu et al., 2020).

Although classified as non-essential for normal cells, recent data indicate that multiple tumours require high glutamine availability and are severely inhibited by its depletion (Wise et al., 2008), making them virtually addicted to this amino acid (Zhang et al., 2017). Glutamine metabolism involves its uptake via SLC1A5, followed by the glutaminolysis process, by which the enzyme glutaminase (GLS) generates glutamate and ammonium (Figure 1). This process supports cancer cell proliferation in multiple ways: 1) via generation of mitochondrial tricarboxylic acid (TCA) cycle intermediates, which are involved in ATP production and lipid synthesis through anaplerosis (Chiu et al., 2014); 2) via glutathione (GSH) synthesis for redox regulation, either acting as a substrate for glutamate-cysteine ligase (GCL) or facilitating cysteine uptake through the cysteine/glutamate transporter xCT (also known as SLC7A11); 3) via purine and pyrimidine biosynthesis, acting as nitrogen donor (DeBerardinis et al., 2007); 4) via NEAA synthesis: glutamate can be transformed into proline through a series of reductive steps, into aspartate, alanine, serine and glycine via transaminases and into asparagine via asparagine synthetase (ASNS).

**FIGURE 2:**
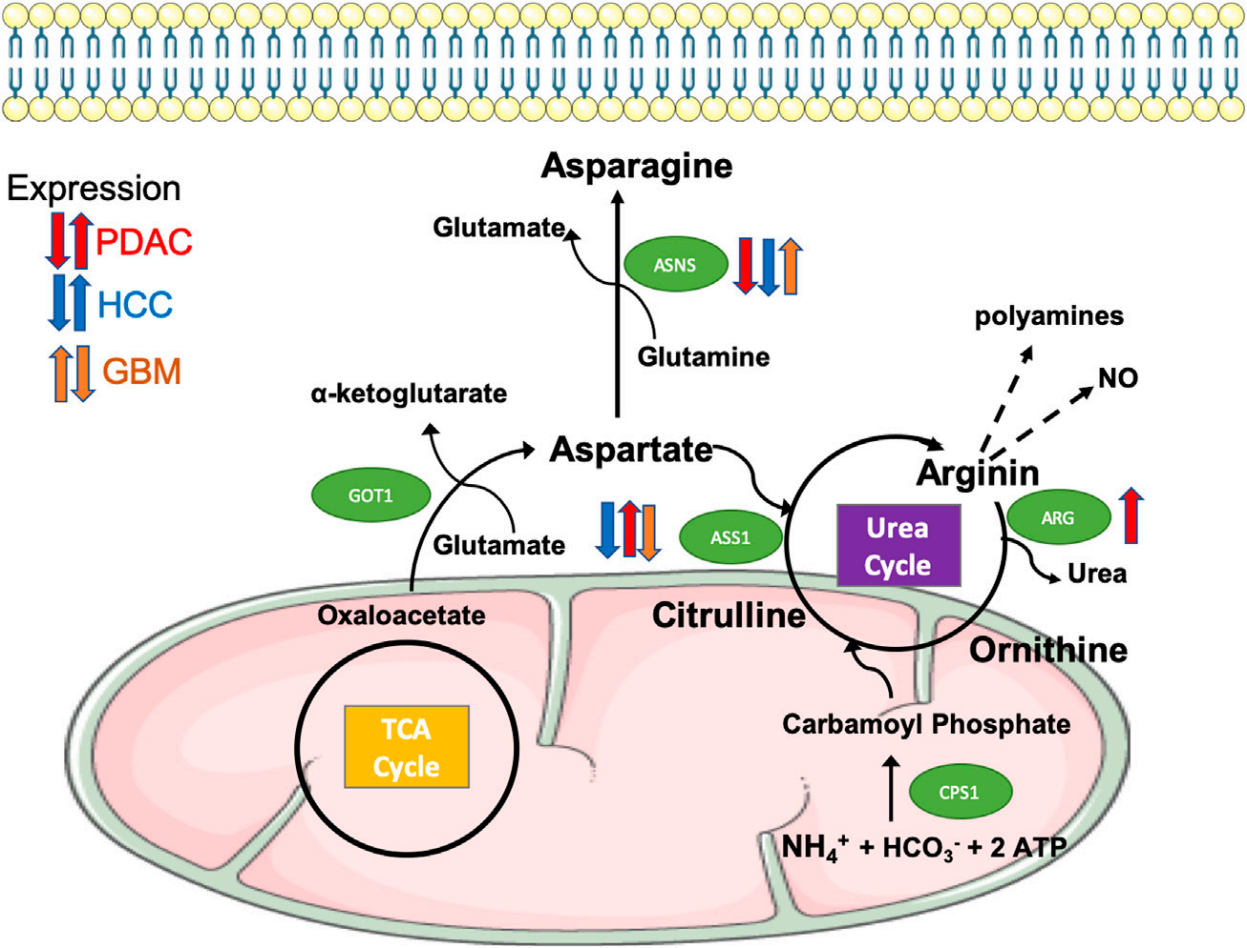
Differential expression of enzymes regulating the biosynthesis and metabolism of asparagine and arginine in aggressive tumours. The amino acid aspartate is synthesised from oxaloacetate, a TCA cycle intermediate, and from glutamate via the transaminase GOT1. Aspartate is then used in the biosynthesis of asparagine mediated by the enzyme ASNS (Asparagine synthetase), enters the urea cycle to contribute to the synthesis of arginine via ASS1 (argininosuccinate synthase 1), or can provide its amino group as free ammonia that builds carbamoyl phosphate via CPS1, thus initiating the urea cycle. Arginine is further metabolised by ARG (arginase, isoforms 1 and 2) to produce urea and ornithine, thus completing the urea cycle, or to polyamines and nitric oxide, both of them implicated in cancer cell aggressiveness through chromatin remodelling and cell signalling. As indicated, the expression of ASNS, ASS1 and ARG is differentially dysregulated in PDAC (red arrows), HCC (blue arrows) and GBM (orange arrows).

One of the main by-products of glutaminolysis is ammonium, which needs to be detoxified in the urea cycle (Morris, 2002). This pathway involves several enzymes, such as argininosuccinate synthetase (ASS) and arginase (ARG). The latter catabolises arginine into urea and ornithine (Figure 2). Additionally, arginine is used as a carrier of nitrogen, whereas arginine-derived polyamines alter gene expression by modulating global chromatin structure and cancer cell proliferation (Pegg, 2009).

The metabolism of the EAA methionine involves the methionine cycle and related pathways, such as transsulfuration, that synthesises GSH for methylation reactions, especially important in cancer. Indeed, methionine is converted into S-adenosylmethionine (SAM, the main methyl donor) in a reaction catalysed by methionine adenosyl transferases (MAT) (Kaiser, 2020), which contributes to nucleotide biosynthesis, necessary for cancer cell proliferation. Methionine can also be used directly as a substrate in polyamine synthesis.

Another EAA, tryptophan, follows a metabolic fate that has acquired great visibility in the last years due to its implication in cancer immunotherapy: kynurenine produced from tryptophan by the enzyme indoleamine 2,3-dioxygenase (IDO) induces immunosuppression (Fallarino et al., 2003; Greene et al., 2019) by binding to and activating the transcription factor aryl hydrocarbon receptor (AhR) (DiNatale et al., 2010; Opitz et al., 2011). This impairs the ability to target and eliminate cancer cells of immune-tolerant dendritic cells (DCs) and regulatory T cells (Mezrich et al., 2010).

## Nutrient Starvation in Pancreatic Ductal Adenocarcinoma

### Metabolism in Pancreatic Ductal Adenocarcinoma Cancer (Stem) Cells

The existence of CSCs in PDAC was initially demonstrated in 2007 (Hermann et al., 2007; Li et al., 2007). They were identified by combining surface markers such as CD44^+^CD24^+^ESA^+^ (Li et al., 2007) or CD133^+^ CXCR4^+^ (Hermann et al., 2007), which help to isolate cancer cell subpopulations with increased self-renewal and tumourigenicity, capable of giving rise to differentiated progenies. Cells with elevated metastatic potential have also been identified using CD133^+^ CXCR4^+^. CSCs in PDAC could be originated either by local stem cells suffering malignant transformation or by differentiated cells acquiring stem-like abilities (Wang et al., 2009). Besides the well-known developmental pathways such as Hedgehog (Li et al., 2007) or Nodal/activin (Lonardo et al., 2011), it has been demonstrated that specific metabolic traits also play a key regulatory role in maintaining the pluripotency and tumourigenic potential of PDAC CSCs (Sancho et al., 2015; Sancho et al., 2016). Indeed, PDAC CSCs are fundamentally oxidative as opposed to their glycolytic differentiated counterparts, a phenotype governed by balance in expression between the oncogene c-MYC and the mitochondrial biogenesis factor PGC-1alpha (Sancho et al., 2015). Precisely, mitochondrial homeostasis in PDAC CSCs is controlled throughout the different stages of the mitochondria lifecycle from mitochondrial biogenesis, via PGC-1alpha upregulation (Sancho et al., 2015), fission, by increasing DRP-1 expression and activity (Courtois et al., 2021), and mitophagy, in a process dependent on the post-translational modification known as mitochondria ISGylation (Alcalá et al., 2020). Blocking any of these processes severely impacts PDAC stemness and tumour-initiating capacity.

In general, cellular metabolism plays a crucial role in tumour progression in PDAC. Indeed, pancreatic tumours are characterised by a desmoplastic stroma that represents up to 90% of the tumour volume. The extensive fibrosis and matrix deposition prevent proper PDAC tumour vascularisation, generating a hypoxic microenvironment with limited nutrient availability. To thrive in this environment, PDAC cells develop a distinct metabolic programme generally characterised by increased glucose and glutamine uptake and metabolism, lipid and protein scavenging, and autophagy to recycle cellular components (Espiau-Romera et al., 2020). Interestingly, extracellular matrix (ECM) proteins, such as collagen, represent an alternate source of energy for PDAC cells, providing proline that feeds the TCA cycle, thus helping PDAC cells to cope with nutrient deprivation (Olivares et al., 2017). Additionally, stromal cells provide essential nutrients to cancer cells, such as TCA cycle components, lipids or amino acids; these metabolites are released extracellularly either directly or carried by extracellular vesicles. These alternative mechanisms create complex metabolic networks amongst the different resident cell types that fuel PDAC cell proliferation, survival and metastasis (Sousa et al., 2016; Zhao et al., 2016; Banh et al., 2020). Additionally, this metabolic interplay also occurs between functionally distinct cancer cells: for instance, glycolytic differentiated tumour cells could provide oxidative CSCs with end-products of glycolysis, such as lactate (Sancho et al., 2015). Theoretically, the metabolic particularities of PDAC CSCs may reflect a proficient adaptation to their nutrient-deprived microenvironment. Indeed, due to their increased mitochondrial activity, they could produce NADH and FADH_2_ used to synthesise ATP in their mitochondria from a wide range of substrates: glycolysis end-products such as lactate and acetyl-CoA derived from glucose, lipids or ketone bodies and several amino acids.

### Glucose Starvation

As mentioned above, the PDAC microenvironment is essentially hypoxic due to the lack of vascularisation, which favours metabolic reprograming towards glycolysis (Cao et al., 2019). Additionally, PDAC driver mutations on K-RAS and TP53 further enhance the expression of glycolytic enzymes and transporters, such as GLUT1, hexokinase 1 and 2 (HK1, HK2) and lactate dehydrogenase A (LDHA). Globally, PDAC metabolic reprogramming improves survival in presence of low glucose levels (Bryant et al., 2014), by enhancing glycolysis, which then fuels the anabolic branches of PDAC metabolism to provide the cancer cells with building blocks for proliferation (Ying et al., 2012). Accordingly, a glycolytic gene expression signature is related to bad PDAC prognosis, invasiveness and metastatic onset (Espiau-Romera et al., 2020; Tian et al., 2020). In fact, samples from metastatic PDAC showed an important enrichment of a glycolysis-related gene signature (Chaika et al., 2012). In addition, epithelial-to-mesenchymal transition (EMT) driven by the transcription factor SNAIL, which promotes cancer metastasis, induced a transcriptional program that enhanced glucose uptake and lactate production (Liu et al., 2019). As predicted from gene expression studies, glycolysis inhibition by the glucose mimetic 2-deoxyglucose (2-DG) (Roy et al., 2015) or lactate eflux inhibition by knocking down the monocarboxylate transporters (MCT) impaired PDAC invasiveness (Kong et al., 2016).

At the same time, most PDAC CSCs are mainly oxidative and could use a wider range of endogenous substrates to fuel their TCA cycle, making them resistant to, not only glucose, but general metabolite deprivation. Indeed, CD133^+^ cells from a patient-derived xenografts (PDXs) panel showed resistance to glucose and glutamine deprivation *in vitro*, while most of the CD133^−^ differentiated counterparts died in such conditions (Sancho et al., 2015). Interestingly, the glucose-dependence of differentiated PDAC cells can be used as a CSC enrichment method *in vitro* (Valle et al., 2020). Indeed, providing galactose instead of glucose as carbon source sharply decreases glycolysis and forces cells to engage mitochondrial OXPHOS to obtain energy and survive. While CD133^+^ PDAC cells were able to proliferate normally under these conditions, CD133^−^ cells suffered cell death, thus increasing the percentage of CSCs. Furthermore, metabolic stress triggered by galactose containing media favoured CSC identity acquisition by a small percentage of non-CSCs, highlighting the close connection between OXPHOS metabolism and stemness in PDAC.

### Amino Acid Starvation

PDAC tumours have developed different strategies to counteract the lack of amino acids in their nutrient-deprived environment. On the one hand, PDAC cells can uptake individual amino acids from plasma (Tumas et al., 2019) or secreted by different stromal cells (Meyer et al., 2016; Sousa et al., 2016; Banh et al., 2020; Zhu et al., 2020) through membrane transporters like L-type amino acid transporter 1 (LAT1) or cystine/glutamate exchanger (SLC7A11/xCT) (Figure 1). For this reason, the expression of amino acid transporters has been associated with chemoresistance and poor prognosis (Altan et al., 2018; Daher et al., 2019). On the other hand, PDAC cells capture and degrade external proteins such as albumin or collagen through macropinocytosis (Davidson et al., 2017; Olivares et al., 2017), representing an essential source of amino acids to sustain central carbon metabolism. Additionally, whole-body protein breakdown is an early event in PDAC development, further intensified as the disease progresses and, especially, under conditions of terminal cachexia (Holmstrom and Olive, 2014).

#### Essential Amino Acids

##### Branched-Chain Amino Acids

High plasma concentration of branched-chain amino acids (BCAAs) leucine, isoleucine and valine is associated with PDAC risk (Mayers et al., 2014; Maertin et al., 2017) and can be considered an early event of PDAC development (Tumas et al., 2019). Although mouse models of PDAC show decreased BCAA levels in later stages of tumour development (Mayers et al., 2016), human PDAC cell lines support fatty acid synthesis via BCAAs, which sustains *in vitro* proliferation (Lee et al., 2019). Indeed, BCAA catabolism inhibition through downregulation of branched-chain keto acid dehydrogenase E1 subunit alpha (BCKDHA) or branched-chain amino acid transaminase 2 (BCAT2) expression significantly impaired clonogenicity, suggesting BCAAs have a supportive role in PDAC self-renewal (Lee et al., 2019).

##### Tryptophan

Very recently, tryptophan metabolism via the enzyme IDO1 has been proven to serve as one-carbon unit for nucleotide synthesis, substituting serine and glycine and, subsequently, supporting tumour growth in genetically engineered PDAC mouse models (Newman et al., 2021). Interestingly, IDO1 expression was higher in anchorage-independent conditions via c-Jun N-terminal kinase (JNK) activation, suggesting that this pathway may be stimulated under CSC-enriching conditions. In addition, the release of formate, a by-product of tryptophan metabolism, by cancer cells can be used by stromal cells to support their own nucleotide synthesis (Newman et al., 2021), representing another example of metabolic crosstalk within the PDAC microenvironment.

#### Non-essential Amino Acids: Conditionally Essential Amino Acids

Lately, different reports have demonstrated that PDAC cells show heterogeneous requirements for specific NEAAs, becoming CEAAs.

##### Alanine

Although alanine requirement in PDAC cells is heterogeneous, since they combine *de novo* synthesis and uptake from the environment at different proportions (Parker et al., 2020), it has been shown that exogenous alanine supplementation supports proliferation and tumour growth in nutrient-deprived conditions (Sousa et al., 2016; Parker et al., 2020), creating a metabolic crosstalk within the tumour microenvironment. Alanine is mainly supplied by pancreatic stellate cells (PSCs), which secrete alanine through different transporters, including SLC1A4, while PDAC cells upregulate the SLC38A2 to promote alanine uptake (Parker et al., 2020). Exogenous alanine will be used to produce pyruvate in order to fuel the TCA cycle, thus decreasing PDAC cells dependence on glucose and serum-derived nutrients. Interestingly, alanine uptake via SLC38A2 supports tumour cells clonogenic and tumour-initiating potential even in nutrient-rich conditions, highlighting another vulnerability of PDAC cells with enhanced tumourigenic potential.

##### Arginine

It has been shown that arginine catabolism by the enzyme arginase 2 (ARG2) supports PDAC tumour growth, specially in an obesity context (Zaytouni et al., 2017). ARG2 is overexpressed in obese mouse models and its expression level is positively correlated with body mass index in PDAC patients. In fact, considering the enhanced protein and amino acid catabolism carried out by pancreatic cancer cells, an increased ARG2 expression and activity could represent a mechanism to detoxify excessive nitrogen through the urea cycle (Figure 2). Accordingly, ARG2 knockdown inhibits tumour growth, severely impairing tumour initiation in obesity conditions (Zaytouni et al., 2017). Several studies demonstrated the implication of arginine in PDAC metastasis. Indeed, arginine deprivation via arginine deiminase (ADI-PEG 20) treatment inhibited the expression of the EMT transcription factors SNAIL, SLUG and TWIST and impaired pancreatic cancer cells adhesion and invasion (Wang et al., 2020). Additionally, arginine promotes PDAC invasiveness via nitric oxide production (Wang et al., 2016).

On the other hand, the enzyme argininosuccinate synthetase 1 (ASS1), that generates the precursor to arginine biosynthesis, has been proposed as a negative PDAC prognostic value (Kim et al., 2020). When arginine deprivation by ADI-PEG was combined with histone deacetylase (HDAC) inhibitors, it impaired clonogenicity and anchorage-independent cell growth in PDAC cell lines (Kim et al., 2020). Together with the report mentioned above, these works suggest that arginine deprivation bears potential to impair PDAC stem-related properties.

##### Asparagine

Asparagine can be considered a CEAA for PDAC. In fact, the expression of the enzyme ASNS, which converts aspartate and glutamine into asparagine and glutamate, is underexpressed or undetectable in the great majority of PDAC patient samples (Figure 2) (Dufour et al., 2012). Indeed, treatment with exogenous L-asparaginase to deplete asparagine concentration reduced PDAC cell lines proliferation *in vitro* and *in vivo* (Dufour et al., 2012). Moreover, further works suggest that a combinatorial approach would effectively target the amino acid metabolism. This is due to the fact that PDAC cells respond to asparagine or glucose starvation by activating the ATF4 transcription factor, via mitogen-activated protein kinase or via AMP-activated protein kinase (AMPK) respectively, to upregulate ASNS (Cui et al., 2007; Pathria et al., 2019). Importantly, ASNS upregulation was responsible for chemoresistance under glucose-starvation conditions, which may compromise the efficacy of this treatment.

##### Cysteine

Several reports have highlighted the critical role of cysteine uptake, specifically in its oxidised form (cystine) via the cystine/glutamate exchanger SLC7A11, which supports tumour initiation and progression in PDAC (Daher et al., 2019; Badgley et al., 2020; Mukhopadhyay et al., 2021). Indeed, SLC7A11 genomic disruption, either via CRISPR-Cas9 in human PDAC cells or in genetically engineered mice, induced cell death via ferroptosis, associated to low levels of intracellular reduced glutathione (GSH), severely impairing PDAC cells clonogenic and tumourigenic potential (Daher et al., 2019; Badgley et al., 2020). Importantly, *in vitro* SLC7A11 transporter inhibition also sentitised cancer cells to chemotherapy, further highlighting the importance of GSH biosynthesis, fuelled by cysteine uptake in this case, for stemness-related properties in PDAC (Jagust et al., 2020).

##### Glutamine

The NEAA glutamine is essential for PDAC cancer cells since it participates in crucial metabolic processes, such as lipid biosynthesis, TCA anaplerosis and redox balance through NAPDH generation and GSH biosynthesis (Liang et al., 2016; Halbrook and Lyssiotis, 2017). In this tumour type, glutamine is metabolised through a non-canonical pathway driven by K-RAS or MYC oncogenes, mediated by transaminases, such as aspartate aminotransferase (Son et al., 2013). Interestingly, glutamine metabolism could partially compensate the ATP drop subsequent to glucose deprivation in MUC1 overexpressing cells (Gebregiworgis et al., 2017).

Recently, it has been described that tumour-initiating cells from the genetically engineered KPC PDAC mouse model upregulate the glutamine transporter ASCT2 and promote its localisation in the plasma membrane via the tetraspanin CD9, enhancing glutamine uptake that fuels tumour initiation and growth (Wang VM. et al., 2019). Partially contradictory data can be found in the literature regarding the effects of glutamine deprivation on PDAC CSCs. On the one hand, CD133^+^ cells isolated from different PDAC PDXs were resistant to glutamine deprivation in terms of apoptosis, when compared with their CD133^-^ counterparts, although no functional data on CSC properties, such as self-renewal, was reported in this case (Sancho et al., 2015). On the other hand, “side population” cells (representing CSCs) and spheroids isolated from pancreatic cell lines were strongly affected by glutamine deprivation, which impaired self-renewal and induced apoptosis due to dysregulation of their redox state (Li et al., 2015; Liao et al., 2017). Furthermore, mitochondrial glutamine transporter ASCT2 knockdown diminished clonogenicity and sensitised PDAC cell lines and PDXs to gemcitabine treatment through impaired reactive oxygen species (ROS) scavenging by GSH (Yoo et al., 2020), further supporting the Gln-GSH axis crucial role in maintaining stemness-related functions in PDAC.

Glutamine deprivation leads to EMT and metastasis in PDAC cells (Recouvreux et al., 2020). Indeed, nutrient stress leads to a MEK/ERK/ATF4 pathway activation, ultimately upregulating the EMT master regulator SLUG, responsible for EMT induction, invasion and survival under glutamine deprivation conditions (Recouvreux et al., 2020).

##### Proline

Proline has been described as a CEAA for a PDAC cell lines subset unable to synthesise proline from glutamate and orthinine (Sahu et al., 2016). For that reason, under proline deprivation conditions, cell lines that depend on this amino acid display constant endoplasmic reticulum stress and deregulated mTORC1-4EBP1, ultimately leading to lower clonogenicity and tumourigenicity (Sahu et al., 2016). Under glucose and glutamine deprivation, PDAC cells can degrade collagen I and IV from the extracellular matrix, uptake fragments via macropinocytosis and then metabolise the incorporated proline as alternative nutrient source to support cancer cell metabolism (Olivares et al., 2017). Under such conditions, the enzyme proline oxidase (PRODH1) catabolises proline to support survival and, crucially, clonogenic and tumourigenic potential. These reports suggest an important link between proline metabolism and tumour-initiating abilities in PDAC cells, underpinning an exploitable metabolic vulnerability for CSC targeting.

##### Serine

Although initially described as NEAA for PDAC cells in the genetically engineered KPC mouse model (Maddocks et al., 2017), further studies have shown that around 40% of human PDAC cell lines depend on exogenous serine to proliferate (Banh et al., 2020). These cells are not able to upregulate crucial genes from the serine biosynthesis pathway (SBP), such as phosphoglycerate dehydrogenase (PHGDH) and phosphoserine aminotransferase 1 (PSAT1), making them dependent on serine released by tumour-infiltrating nerves to the microenvironment (Banh et al., 2020). While serine participates in several metabolic pathways, such as GSH biosynthesis or the folate cycle, serine deprivation in PDAC cells that depend on this amino acid only affected translation of UCC and UCU mRNA codons and, interestingly, increased the selective translation and secretion of nerve growth factor to facilitate nerve recruitment into the tumour. Whether CSCs from serine-dependent PDAC tumours maintain the same serine requirements as their differentiated counterparts remains to be investigated.

## Hepatocellular Carcinoma

### Role of Cancer Stem Cells in Hepatocellular Carcinoma

The liver is the major site for metabolic processes in the body, including blood detoxification, bile production for fat breakdown in the digestive track, glucose storage in the form of glycogen, and the amino acid precursors synthesis. Hepatocytes make up to 85% of the total liver mass and are responsible for most metabolic processes. HCC, the liver cancer that originates from hepatocytes, dysregulates a large number of metabolic processes to fuel tumourigenesis (Nwosu et al., 2017). HCC is a genetically heterogeneous and complex type of tumour. So far, molecular data have poorly contributed to explain the clinical variability of the disease and have not resulted into prognostic or predictive biomarkers (El-Serag, 2011). HCC is the most common liver cancer and one of the leading causes of cancer-related deaths, with increasing prevalence globally (Dhanasekaran et al., 2012; Wang et al., 2018). Sorafenib, regorafenib and lenvatinib are drugs currently used as treatment for HCC. Whether combined or not with radiation therapy and chemotherapy, they have been shown to enhance survival rates in individuals with non-resectable HCC (Jindal et al., 2019; Faivre et al., 2020). Unfortunately, the clinical benefits achieved are limited and patients develop rapid drug resistance and tumour recurrence (Jindal et al., 2019; Faivre et al., 2020). In the context of HCC, CSCs are termed liver cancer stem cells (LCSCs) and are associated to metastasis and tumour relapse after developing drug resistance (Li and Zhu, 2019). Regarding their origin, a widely accepted theory proposes that the inflammatory microenvironment that commonly characterises HCC, whatever its cause (chronic viral infection, alcoholic or non-alcoholic fatty liver disease or long-term exposure to toxicity), leads to the proliferation of stem cells with genetic or epigenetic alterations, facilitating their transformation from normal liver stem cells to LCSCs (Wang et al., 2018; Wu et al., 2020). There are different surface markers, including epithelial cell adhesion molecule (EpCAM), CD133, CD90, CD13, CD44, CD24, CD47, oval cell marker (OV-6), aldehyde dehydrogenase (ALDH), keratin 19 (K19), cKit and ATP binding cassette subfamily G member 2 (ABCG2), which influence the activation of signalling pathways, cell phenotypes and therapeutic drugs resistance in LCSCs (Liu et al., 2020).

### Glucose Starvation

LCSCs have adapted to overcome oxygen and nutrient deficiency that commonly characterises HCC tumour microenvironment (Tsuchiya and Shiota, 2021). CD133 is a surface marker expressed in LCSCs encoded by the PROM1 gene. Other cells like neurons and bone marrow progenitor cells also express CD133, but in the context of HCC, it is suggested that CD133 performs regulatory functions required in maintaining LCSCs undifferentiated status (Tsuchiya and Shiota, 2021). Treatment with a CD133 monoclonal antibody resulted in cell death in hepatoma LM3, HepG2, Hep3B and Huh-7 cells, especially under low glucose conditions (Chen et al., 2013a). It also inhibited the formation of spheroids, colonies and xenograft tumours. These effects were attributed to the autophagy blockage and cell death increase. Therefore, targeting CD133 under low glucose conditions may represent a potential therapeutic approach for HCC (Chen et al., 2013b). It is important to highlight that autophagy is a conserved mechanism for degradation of intracellular material in lysosomes and metabolite recycling, that plays a key role in stem cell maintenance, self-renewal and various cell differentiation processes (Chen et al., 2018). Another study also demonstrated that CD133 regulates autophagy and glucose uptake in response to low glucose conditions, which is vital for cell survival and tumour growth (Chen et al., 2013a). In addition, authors showed that, under glucose starvation conditions, CD133 expression stimulated the formation of autophagosomes and increased glucose uptake and ATP production. In contrast, silencing of CD133 reversed these activities and reduced xenograft tumour formation in NOD/SCID mice. Therefore, this work demonstrated that autophagy regulation and glucose uptake by HCC cells expressing CD133 is involved in cell survival and may be essential for LCSCs to survive in a nutrient-deficient tumour microenvironment (Chen et al., 2013a). Additional data have suggested a regulatory role of HBP on CD133^+^ LCSCs under low glucose conditions (Lin et al., 2016). Under high glucose conditions, HBP inhibition reduced the proportion of the CD133^+^ LCSCs subpopulation and CD133 expression (Lin et al., 2016). In addition, under low glucose conditions and in the presence of GlcNAc, which is commonly used to increase HBP, CD133^+^ LCSCs were rescued. Altogether, these data indicated that the HBP may coordinate with the glycolytic pathway to regulate CD133 expression in hepatoma cell lines, thus maintaining the CSC-like phenotype (Lin et al., 2016). Interestingly, metformin (known to perturb mitochondrial OXPHOS and to activate AMPKs via AMP accumulation) inhibited proliferation, migration and invasion in LCSCs, effect maximised by glucose deprivation (Ferretti et al., 2019). Hence, metformin arises as a potent migration and invasion inhibitor in HCC cells, and therefore, a combined therapy for HCC would be an effective strategy.

### Amino Acid Starvation

#### Essential Amino Acids

##### Branched-Chain Amino Acids

In contrast to PDAC, BCAAs have proven their effects in inhibiting liver cancer cell proliferation and neovascularisation. Consequently, they may benefit patients with HCC (Tajiri and Shimizu, 2018). Importantly, BCAAs also induce the differentiation of EpCAM^+^ LCSCs, sensitising them to chemotherapy (Nishitani et al., 2013). This effect was mediated by mTORC1 activation upon treatment with high doses of BCAA, leading to Wnt/β-catenin pathway inhibition. As a result, LCSCs lost EpCAM expression and significantly decreased their tumourigenic potential and chemoresistance.

##### Methionine

In the liver, methionine is converted into SAM, the most important cofactor for transmethylation reactions (Lu and Mato, 2012), via the enzyme methionine adenosyl-transferase MAT1A, mainly expressed in adult liver. Deactivation of the isoform type 1 of MAT1A causes HCC development (Martínez-Chantar et al., 2002). Moreover, liver carcinogenesis has been associated with a switch between MAT1A and MAT2A (mostly expressed in foetal liver and extrahepatic tissues), resulting in a less efficient methionine metabolism that enhances HCC cell proliferation and genomic instability, as it is the case with global DNA hypomethylation (Frau et al., 2013). Not surprisingly, the pro-tumourigenic effects caused by SAM deficiency can be inhibited by reconstituting its normal levels via exogenous administration (Wang et al., 2017).

##### Tryptophan

The expression level of IDO1 positively correlates with the occurrence of distant metastases in HCC (Opitz et al., 2020). Furthermore, lack of IDO1 resulted in reduced HCC formation and Foxp3^+^ T-reg cells infiltration in an IDO1 knockout mouse model (Shibata et al., 2016).

Immune checkpoint blockade with anti-CTLA-4 and anti-PD1 antibodies has shown promising results in HCC, but some HCC patients developed resistance to immune checkpoint inhibitors by upregulating IDO1. Therefore, combining immune checkpoint and IDO inhibitors could improve the HCC treatment outcome (Brown et al., 2018).

Moreover, a key enzyme involved in tryptophan degradation, kynurenine 3-monooxygenase, was highly expressed in HCC compared to normal tissue. *In vitro* studies have shown that it promotes HCC cells proliferation, migration, and invasion (Jin et al., 2015).

#### Non-Essential Aminoacids: Conditionally Essential Amino Acids

##### Arginine

HCCs are often deficient in ASS1 (Figure 2) and depend on exogenous arginine for cell proliferation, growth, and survival (Ensor et al., 2002). Interestingly, early studies had already shown that arginine depletion in hepatoma using arginine deiminase (ADI) purified from *Mycoplasma arginini* and *Mycoplasma hominis* resulted in hepatoma cell lines growth inhibition (Takaku et al., 1995).

##### Asparagine

Similar to the situation observed in PDAC, the asparagine biosynthesis enzyme ASNS expression is downregulated or even lost in malignant HCC cases (Figure 2). In fact, ASNS re-expression can suppress tumourigenic phenotypes, confirming its tumour-suppressing role (Zhang et al., 2013). Consequently, treatments using asparaginase have shown to arrest proliferation and induce apoptosis in HCC (Tardito et al., 2011).

##### Glutamine

Lower plasma levels of glutamine have been observed in HCC patients due to the increased uptake by tumours via glutamine transporter ASCT2 upregulation in HCC cells (Sun et al., 2016), which has also been observed in PDAC (Wang VM. et al., 2019). Interestingly, the enzyme glutamine synthetase (GS) is overexpressed and represents a diagnostic marker for HCC (Di Tommaso et al., 2007). In normal human and rodent livers, GS is expressed in early hepatocyte precursors and its hepatocyte expression is higher during regenerative states, such as chronic hepatitis B and C, focal nodular and peritumoural hyperplasias, and in some neoplasms, including hepatocellular adenoma and hepatocellular carcinoma (Fleming and Wanless, 2013).

Tumours with activating mutations in the gene that encodes for β-catenin (CTNNB1) segregate as a group with distinctive clinical and genetic characteristics (Cieply et al., 2009). These tumours show constitutive activation of the canonical Wnt pathway, display distinctive histology (Dal Bello et al., 2010) and overexpress a number of target genes functionally linked to amino acid metabolism (Boyault et al., 2007). Indeed, these tumours increase GS expression, resulting in higher intracellular glutamine levels that activate mTORC1 and lead to an enhanced protein translation, which in turn supports cell growth and proliferation (Adebayo Michael et al., 2019). Mutations in other upstream components of the Wnt pathway, such as the AXIN1 gene, may have the same functional effect (White et al., 2012).

Besides GS, it has been described that an increased GLS1 expression is associated with stemness and advanced clinicopathological features in HCC. In fact, GLS1 depletion results in higher ROS levels and Wnt/β-catenin pathway inhibition (Li et al., 2019), promoting differentiation.

##### Glycine

The enzyme glycine decarboxylase (GLDC), which belongs to the glycine cleavage system, is downregulated in HCC cells to support tumour progression and metastasis. On the one hand, GLDC downregulation promoted intracellular ROS levels accumulation, while reducing the GSH/GSSG ratio in HCC cells, which enhanced ROS-induced cell migration via cofilin stabilisation (Zhuang et al., 2018). On the other hand, ROS accumulation following GLDC downregulation, modulated autophagy levels in metastatic HCC, a necessary process to sustain their migratory capacity (Zhuang et al., 2019).

##### Proline

Hydroxyproline, the direct proline derivative, correlates with HCC pathogenesis; HCC tumours show increased proline consumption and hydroxyproline accumulation, which promotes HCC progression and resistance to Sorafenib by modulating hypoxia-inducible factor 1-alpha (Tang et al., 2018).

##### Tyrosine

Tyrosine is mainly degraded in the liver to produce gluconeogenesis and ketogenesis intermediates or precursors. It was already described in the 1980s that HCC patients had elevated serum tyrosine levels (Watanabe et al., 1984), which could indicate tyrosine metabolism deregulation. It was also described that tyrosine aminotransferase (TAT) downregulation contributes to HCC progression (Fu et al., 2010). Not only TAT expression is downregulated in HCC, but also that of other genes involved in tyrosine catabolism, such as 4-hydroxyphenylpyruvic acid dioxygenase, homogentisate 1,2-dioxygenase, and GTSZ1. Their low expression correlates with poor prognosis (Nguyeni et al., 2020). In contrast, a different study showed that CD13^+^ LCSCs had negligible glycolytic activity, and that their viability did not depend on glucose or glutamine, but on tyrosine, as tyrosine deprivation induced apoptosis. High tyrosine consumption in CD13^+^ cells is concomitant with higher expression levels of enzymes involved in tyrosine degradation compared to CD13^−^ cells, rendering CD13^+^ addicted to tyrosine. Interestingly, targeting tyrosine metabolism by nitisinone eliminates CD13^+^ LCSCs and enhances the anti-tumourigenic effects of 5-fluorouracil in an *in vivo* setting (Sun et al., 2019).

## Glioblastoma

### Glioblastoma and Cancer Stem Cells

GBM develops in the glia of the human brain and represents the most common and malignant type of brain tumours (Prager et al., 2019; Waker and Lober, 2019). A positive aspect is that its prevalence is estimated at 1/100 000 and it is usually diagnosed in 45- to 70-year-old patients. However, the prognosis of patients is poor, surviving only 12 months after diagnosis. This survival time can be slightly increased after chemotherapy with temozolomide, when combined with radiotherapy and surgery. Temozolomide causes double-stranded DNA breaks, which lead to cell cycle arrest in the G2/M phase and eventually to cell death (Nagel et al., 2017).

GBM belongs to the gliomas, a broader class of brain tumours, with three subtypes: classical, mesenchymal and proneural. These subtypes are characterised by their genetic mutations, cell of origin and invasiveness. The classical GBM subtype usually contains phosphatase and tensin homolog (PTEN) mutations and epidermal growth factor receptor (EGFR) amplification. The proneural subtype is characterised by phosphoinositide-3′-kinase (PI3K) mutations and platelet-derived growth factor receptor alpha (PDGFRA) amplifications (Prager et al., 2019; Waker and Lober, 2019). The mesenchymal GBM subtype typically contains loss-of-function mutations in the tumour suppressor genes *p53*, *PTEN* and NF1 (Prager et al., 2019; Waker and Lober, 2019). It is also the most aggressive due to its high angiogenic and invasive capacities (Saito et al., 2019). The three subtypes are mainly determined by three potential GBM cells of origin: 1) the neural stem cells (NSCs), with a high regenerative plasticity and development potential (Jacques et al., 2010); 2) the NSC-derived astrocyte progenitors that generate the mature astrocytes through symmetric division in adults (Chow et al., 2011); and 3) the oligodendrocyte precursor cells, which represent the main dividing cell population in the adult brain (Rebetz et al., 2008). Common features of the three GBM subtypes are their high proliferation rate and chemoresistance. They are able to form secondary tumours inside and, more rarely, outside the brain (Anderson et al., 2020).

Identifying glioblastoma stem cells (GSCs) is difficult due to the high plasticity of glioblastoma cells, allowing them to shift from a non-GSC state to a GSC state in response to molecules and other cells in their microenvironment (Safa et al., 2015). For instance, nutrient deprivation (Mondal et al., 2018) and hypoxia (Bar et al., 2010) induce metabolic changes that regulate GSCs interconversions. Such metabolic shifts are associated with phenotypic changes, for example an increased self-renewal capacity in GSCs and proliferation rates, as well as the capacity to initiate a tumour *in vivo* that reproduces the cellular complexity of the original tumour (Lathia et al., 2015). In order to distinguish the glioblastoma cells from GSCs, intracellular proteins (SOX2, OLIG2, MYC and NESTIN) and cell surface proteins (CD133, SSEA-1 and CD44) are used as detection and discrimination agents (Suvà et al., 2014; Fiscon et al., 2018). GBM tumours use glucose for both glycolysis and oxidative phosphorylation, and synthesise glutamine and glycine *de novo* from glucose (Maher et al., 2012; Marin-Valencia et al., 2012).

### Glucose Starvation

Glucose metabolism is different depending on the GBM tissue region. In the surrounding normal tissue, glucose is converted to pyruvate through glycolysis and pyruvate enters the TCA cycle producing ATP via oxidative phosphorylation. A different mechanism works in the central region of the tumour, where glucose also produces ATP via glycolysis, but the resulting pyruvate provides mainly lactic acid that accumulates in this region. Lactic acid can be a signalling factor and increases the expression of the H^+^/lactate symporters MCT4 and MCT1. MCT4 effluxes lactic acid to the lateral region of the tumour where MCT1 uptakes it to be part of the mitochondrial oxidative metabolism to maintain the high level of ATP (Duan et al., 2018). In GBM, under low glucose conditions, various mechanisms that promote the survival of tumour cells are activated. It is reported that, on the one hand, cytosolic glutamate dehydrogenase 1 (GDH1) binds to the IKK (inhibitor of NF-κB kinase) complex and converts glutamate to α-ketoglutarate (α-KG). α-KG activates IKKβ and NF-κB signalling, responsible for the overexpression of GLUT1 which promotes glucose uptake, glycolysis and tumour cell survival under glucose deprivation conditions (Wang X. et al., 2019). On the other hand, glucose deprivation creates the perfect environment for xCT-induced cell death, due to increased ROS levels. Glioblastoma cells frequently show a high expression of xCT, the light chain subunit of the x_c_^−^ system, responsible for extracellular cysteine and intracellular glutamate exchange across the plasma membrane (Figure 1). xCT expression correlates with tumour growth and poor survival. In this context, cell density plays an important role because high density inhibits the function of mTOR and consequently increases xCT lysosomal degradation, promoting GBM cell survival (Yamaguchi et al., 2020) and thus protecting tumour cells from glucose deprivation-induced cell death. Moreover, under nutrient deprivation conditions, GSCs upregulate GLUT3 expression (Flavahan et al., 2013). When considering glycolysis and tumourigenesis, PTEN is a relevant factor. This tumour suppressor is frequently mutated or deleted in cancer, having an important role in glucose metabolism via the PI3K/AKT pathway. It has been described that PTEN establishes direct contact with the glycolytic enzyme phosphoglycerate kinase 1 (PGK1), dephosphorylating it and inhibiting its autophosphorylation; ultimately PTEN inhibits glycolysis, ATP production and brain tumour cell proliferation (Qian et al., 2019). Another tumour suppressor associated with glucose metabolism is p53. This protein stimulates apoptosis in GBM under acute glucose metabolism inhibition conditions (Mai et al., 2017). In other cases, glucose levels do not seem to play a major role. AMPK is described as a cellular energy sensor able to regulate the GBM bioenergetics transcriptional programme. In normal cells, glucose starvation activates AMPK, whereas the abundance of glucose reduces its activity. In GSCs, AMPK activity is chronically increased and depends on glucose levels. Consequently, AMPK inhibition reduces GSC viability (Chhipa et al., 2018).

The first step in glucose metabolism is converting glucose into glucose-6-phosphate. Glucose-6-phosphate participates in glycolysis, but it also takes part in the glycosaminoglycan (GAG) synthesis pathway. High levels of GAG are implicated in GBM and the UDP-glucose 6-dehydrogenase (UGDH) is a relevant enzyme for their production. UGDH regulates cell proliferation and migration *in vitro* and its inhibition reduces the amount of GAG and other ECM key components in GBM cells, thus compromising GBM growth (Oyinlade et al., 2018).

In GBM cells, glucose deprivation translates into bioenergetic stress. The exchange of glucose for galactose as carbon source causes glycolytic inhibition and dependence on mitochondrial oxidative phosphorylation. The mitochondrial oxidative phosphorylation results in ATP molecules that provide energy for an optimal cell growth and survival. GSCs are able to easily adapt to the glucose-galactose shift, probably by increasing O_2_ consumption rates and basal respiration to compensate for glycolysis decrease, as well as by enhancing NF-κB-inducing kinase and dynamin-related protein 1-dependent mitochondrial fission (Kamradt et al., 2021).

Interestingly, whether GSCs are dependent on glycolysis or on oxidative phosphorylation has been debated for more than a decade. According to the study by Vlashi et al., in 2011, GSCs were less glycolytic than differentiated glioma cells, concluding that GSCs rely mainly on oxidative phosphorylation. However, when OXPHOS was inhibited, the GSCs could use additional metabolic pathways (Vlashi et al., 2011). More recent studies have shown that GSCs can produce both glycolytic and mitochondrial energy to sustain tumour propagation, and, as described in 2011, GSCs relying on OXPHOS have more plasticity and can switch towards a glycolytic phenotype when needed (Shibao et al., 2018).

Finally, D-galactose is described as a cell senescence inducer through glutamine synthetase signalling (Shen et al., 2014) or by compromising the autophagy flux and mitochondrial functions (Xu et al., 2018). The mechanism of senescence induction by D-galactose implicates the YAP/CDK6 pathway inactivation (Xu et al., 2020).

### Amino Acid Starvation

#### Essential Amino Acids

##### Branched-Chain Amino Acids

The first step of BCAA catabolism generates glutamate and branched-chain ketoacids (BCKAs); the high BCKA excretion levels in GBM cells are impacted by the MCT1 transporter (*SLC16A1*) expression levels. Interestingly, MCT1 is located near BCAT1. The excreted BCKAs are then uptaken by macrophages and reduce the phagocytic capacity of macrophages, which could identify BCKA as a tumour immune suppressor (Silva et al., 2017).

##### Methionine

Studies on cellular methionine uptake by GBM cells have shown a significant uptake when compared to normal human astrocytes (Palanichamy et al., 2016). Interestingly, the expression of the key enzyme for methionine salvage, methylthioadenosine phosphorylase (MTAP), lacks in the majority of GBMs due to MTAP promoter deletion or methylation (Palanichamy et al., 2016). MTAP loss results in an increased methionine uptake. When comparing methionine deprivation effects on GBM cell lines grown under conditions that favour differentiation (monolayer in media with serum) versus the same cell lines grown in stem cell conditions (spheres with a stem cell media composition), it was observed that only when GBM cells were grown under stem cell conditions, methionine was required for neurosphere formation (Zgheib et al., 2019).

##### Tryptophan

The enzyme IDO1 is highly expressed in GBM patients (Zhai et al., 2017). In GBM mouse models where IDO1 expression has been genetically suppressed, a decrease of intra-glioma T-reg accumulation as well as loss of T-cell-mediated survival was observed (Wainwright et al., 2012, 2014). Uptake monitoring of α-[^11^C]-methyl-*L*-Trp (AMT), a tryptophan analogue that allows for tryptophan metabolism quantification, has revealed that higher intratumoural AMT uptake is associated with shorter survival (Kamson et al., 2014; Lukas et al., 2019).

#### Non-Essential Amino Acids: Conditionally Essential Amino Acids

##### Arginine

Due to ASS1 and/or arginosuccinate lyase silencing (Figure 2), 30% of GBM patients are auxotrophic for arginine (Syed et al., 2013; Mörén et al., 2018). The majority of arginine present in the central nervous system comes from its biosynthesis in the kidney and imported from peripheral blood, which renders such GBM patients sensitive to arginine pharmacological depletion (Przystal et al., 2018). Arginine deprivation in GBM cells decreased cell viability, cell motility, invasiveness and adhesion; arginine deprivation resulted in decreased β-actin filament content, and in reduced β-actin arginylation (Pavlyk et al., 2015), shown to be crucial for cell migration and cardiomyocyte contractility (Kurosaka et al., 2012).

##### Asparagine

The ASNS gene is amplified in GBM (Figure 2), correlating with decreased survival (Thomas et al., 2021). Forced ASNS overexpression in GSCs led to a slower basal metabolism and greater plasticity to increase glycolysis or OXPHOS when necessary. The changes in metabolism were concomitant with increased proliferation and invasiveness, as well as with increased radiation resistance due to a higher endurance under cellular and oxidative stress (Thomas et al., 2021). Patients who do not express high levels of ASNS might benefit from being treated with recombinant L-asparaginase, since this treatment induces apoptosis *in vitro*, reduces GBM growth when tumour cells are implanted subcutaneously in mice and enhances ABT263 anti-tumoural effects (a BCL2/BCL-XL inhibitor) (Karpel-Massler et al., 2016).

##### Glutamine

*In vitro* studies have shown that glutamine is used by GBM cell lines to produce TCA intermediates. It is then converted to lactate producing NADPH, which provides energy for fatty acid and nucleotide production (DeBerardinis et al., 2007). In contrast, *in vivo* studies have shown that glucose was preferred over glutamine for oxidative metabolism in GBM (Marin-Valencia et al., 2012). Later, it was shown that GBM cells were indeed not dependent on glutamine, since glutamine deprivation did not always reduce GBM cell proliferation. Interestingly, GCSs have a higher GS expression, rendering their growth independent of glutamine supplementation compared to differentiated GBM cells derived from the same patients. Finally, it was also observed that the stem-like population did not depend on glutamine for anaplerosis (Tardito et al., 2015). According to this study, most glutamine in GBM cells is transformed to glutamate, which does not sustain fatty acids biosynthesis under normoxic conditions (Tardito et al., 2015).

The source of glutamine in GBM cells has been proven to be mainly glucose-derived or produced by the astrocytes in the tumour microenvironment, as injection of labelled glutamine was barely uptaken by GBM cells (Tardito et al., 2015). In a different study, it was shown that patient-derived GSCs cluster in two groups, one that is glucose-dependent and another group that adapts to glucose deprivation, since it can use glutamine for anaplerosis (Oizel et al., 2017). Interestingly, the group of patient-derived GSCs that uptake high glutamine levels, transcriptomically classifies as the GBM mesenchymal subtype, while the glucose-dependent group does not (Oizel et al., 2017). Not surprisingly, tumours generated by mesenchymal GSCs show increased glutamine uptake and conversion to glutamate when compared to non-mesenchymal GSCs (Oizel et al., 2020). In the same study, it was shown that the non-mesenchymal GSCs (which belong to the proneural and classical subtypes) express high levels of GS (Oizel et al., 2020). These studies seem to contradict the work mentioned earlier by Tardito et al. (2015) and Marin-Valencia et al. (2012), in which the authors described that the stem-like population does not depend on glutamine. However, GBM cells had not been classified as mesenchymal or non-mesenchymal in those studies, according to Verhaak et al. (2010), so it could be possible that they did not include any mesenchymal GBM cultures, which can explain the apparent disagreement.

In a more recent study, it was demonstrated that GSCs have different responses to glutaminase inhibition; GSCs that depend on glutaminolysis for cell growth and viability have low expression of the astrocytic glutamate transporters, also known as excitatory amino acid transporters (EAAT), EAAT1 and EAAT2. In such cells, targeting glutaminase leads to intracellular glutamate depletion, triggering the amino acid deprivation response, causing cell death. GSCs that express high EAAT1/EAAT2 levels can be sensitised to glutaminase inhibition by blocking glutamate transport with L-trans-pyrrolidine-2,4-dicarboxylic acid (PDC, Figure 1) (Restall et al., 2020).

Moreover, there is a group of GBM patients with a mutation at the R132 residue of isocitrate dehydrogenase 1 (IDH1), giving rise to a neoenzyme, which produces the oncometabolite 2-hydroxyglutarate (2-HG) from α-KG. In such cases, IDH1 mutant cells depend on glutamine to obtain α-KG. First, glutamine is converted to glutamate via glutaminase and finally it is metabolised to α-KG via transaminases. In addition, 2-HG inhibits BCAA transaminases, BCAT1 and BCAT2, impairing BCAA catabolism and glutamate biosynthesis, which also explains IDH1 mutant cells dependency on glutaminase to obtain glutamate from glutamine (McBrayer et al., 2018).

##### Glutamate

Glutamate is a major cause of brain toxicity associated with glioblastoma growth (Ye and Sontheimer, 1999) and may be responsible for brain edema as well as other tumour-related symptoms. Glutamate also supports GBM cells invasion (Lyons et al., 2007). Interestingly, even though GBM and GSCs express high levels of GLAST, a glutamate-aspartate transporter expressed in astrocytes responsible for glutamate uptake, GSCs release, rather than uptake, glutamate because they lack Na^+^/K^+^-ATPase expression, needed to create the Na^+^ electrochemical gradient necessary for glutamate uptake to take place. Na^+^/K^+^-ATPase overexpression restores glutamate uptake and induces apoptosis in GSCs (Corbetta et al., 2019).

##### Serine

In gliomas, PHGDH expression, the first and rate-limiting step to divert substrates from glycolysis to serine production, was increased while PHGDH silencing reduced proliferation and invasiveness in GBM cells (Liu et al., 2013). In hypoxic conditions PHGDH levels were increased and serine restriction via PHGDH inhibition sensitised GBM cells to hypoxia-induced cell death (Engel et al., 2020).

##### Taurine

Taurine is an amino sulfonic acid not used for protein synthesis, but with several physiological roles as osmotic pressure controller, neuromodulator and immunomodulator (Shen et al., 2021). Taurine is synthesised either from cysteine oxidation via cysteine dioxygenase, which generates cysteinesulfinate that is decarboxylated by cysteinesulfinic acid decarboxylase, or from cysteamine oxidation by cysteamine (2-aminoethanethiol) dioxygenase (ADO) (Ueki and Stipanuk, 2009). ADO expression is significantly higher in GBM when compared to lower grade gliomas and its expression is upregulated in GSCs. Moreover, ADO overexpression promotes glioma stemness via NF-κB signalling (Shen et al., 2021).

## Metabolic Therapeutic Approaches for Cancer Stem Cells Targeting

### Targeting Amino Acids

As inferred from the previous sections, amino acid metabolism represents one of the most promising approaches for targeting CSCs in PDAC and GBM through nutrient deprivation. Efforts have been made to develop therapeutic agents targeting amino acid transport and catabolic/biosynthetic pathway in many different cancer types. In this section we aim to summarise strategies against specific amino acids in our focus cancer types (Table 1). The transporter SLC6A14, which transports 18 of the 20 amino acids including all EAAs, was found to be upregulated in PDAC PDXs, primary tumour tissues and cell lines compared to normal tissue or cells (Coothankandaswamy et al., 2016), but not in GBM and HCC. Interestingly, SLC6A14 inhibition with α-methyltryptophan (Figure 1) induced amino acid starvation in PDAC cells and not only reduced tumour growth and proliferation *in vivo* and *in vitro*, but also CSC-related features such as clonogenicity and invasiveness. The use of such inhibitor could also have promising outcomes when applied to other cancer types.

**TABLE 1:** Clinical trials targeting amino acids.

	Trial ID	Phase	Target aa	Intervention	Status
PDAC	NCT02071862	I	Gln	CB839	Completed
	NCT01523808	I	Asn	GRASPA	Completed Bachet et al. (2015)
	NCT02195180	II	Asn	ERY001 + Gemcitabine or FOLFOX	Completed Hammel et al. (2020)
	NCT03665441	III	Asn	Eryaspase + Gemcitabine/Abraxane or FOLFIRI	Recruiting
	NCT02101580	I/Ib	Arg	ADI-PEG 20 + Gemcitabine/Abraxane	Completed Lowery et al. (2017)
	NCT00739609	I	IDO	Indoximod	Terminated
	NCT02077881	I/II	IDO	Indoximod + Gemcitabine/Abraxane	Completed
	NCT03432676	II	IDO	Epacadostat + Pembrolizumab	Withdrawn
	NCT03006302	II	IDO	Epacadostat + Pembrolizumab + CRS-207 ± Cyclophosphamide/GVAX	Recruiting
	NCT03085914	I/II	IDO	Epacadostat + Pembrolizumab + Chemotherapy (7 different combinations)	Active, not recruiting
GBM	NCT04587830	I	Arg	ADI-PEG20 + radiotherapy and temozolomide	Recruiting
HCC	NCT 01287585	III	Arg	ADI-PEG 20	Completed Abou-Alfa et al. (2018)
	NCT00056992	II	Arg	ADI-PEG20	Completed
	NCT02006030	II	Arg	Phase 2 Trial of ADI-PEG20 + Concurrent Transarterial Chemoembolization (TACE) Versus TACE Alone	Completed
	NCT021020022	I/II	Arg	ADI-PEG20 + modified FOLFOX6	Terminated Harding et al. (2018)
	NCT02101593	I	Arg	ADI-PEG20 + Sorafenib	Completed
	NCT02178722	I/II	IDO	Combining MK-3475 + INCB024360	Terminated
	NCT03277352	I/II	IDO	INCAGN01876 or Epacadostat or Pembrolizumab	Completed

#### Targeting Essential Amino Acids

##### Tryptophan

The administration of indoximod (an oral IDO1 inhibitor) combined with temozolomide has demonstrated synergistic survival benefits in GBM mouse models (Wainwright et al., 2014; Hanihara et al., 2016), which is under investigation in GBM patients (Zakharia et al., 2015, 2016). Moreover, different clinical trials against IDO1 in PDAC and HCC are ongoing.

#### Targeting Non-essential Amino Acids

##### Arginine

Arginine deprivation has been proven to impair clonogenicity and tumour initiation (Zaytouni et al., 2017; Kim et al., 2020), as well as PDAC prometastatic properties (Wang et al., 2020). Interestingly, the amino acid starvation by ADI-PEG 20 also sensitised PDAC cells to gemcitabine treatment (Prudner et al., 2019) and radiotherapy in ASS1-deficient pancreatic cell lines (PANC-1, Miapaca-2, AsPC1, and Capan1) (Singh et al., 2019). The use of ADI-PEG 20 enhanced the survival rate of mice with intracranial GBM and demonstrated therapeutic synergy with temozolomide in tumours expressing ASS1 (Przystal et al., 2018). In HCC, several clinical trials have been performed using ADI-PEG 20 (Table 1) (Izzo et al., 2004; Yang et al., 2010; Harding et al., 2018), showing acceptable safety and initial results demonstrating efficient elimination of detectable plasma arginine in a subset of HCC patients. Yet, a Phase III clinical trial using ADI-PEG 20 as monotherapy did not improve overall survival in a second-line setting for HCC (Abou-Alfa et al., 2018).

##### Asparagine

Considering the low ASNS expression in PDAC patients and the anti-tumoural effects seen in different *in vitro* and *in vivo* preclinical data (Dufour et al., 2012), PDAC treatment with L-asparaginase encapsulated in red blood cells (GRASPA, ERY001, Eryaspase) is under intense testing in clinical trials (Table 1). Indeed, after a phase I trial demonstrating that this treatment was well tolerated by metastatic PDAC patients (Bachet et al., 2015), a phase IIb trial showed improvements in progression-free and overall survival when combined with gemcitabine or FOLFOX in second-line advanced PDAC, irrespective of ASNS expression (Hammel et al., 2020). This last trial provided particularly important data since chemoresistant PDAC cells upregulate ASNS that could compromise the efficacy of the L-asparaginase approach (Cui et al., 2007). Interestingly, L-asparaginase was able to deregulate the redox balance of side population CSC-like cells, significantly affecting their stemness potential (Liao et al., 2017), although the authors of the study attributed these anti-tumoural effects to glutamine deprivation and not to direct impact on asparagine homeostasis.

##### Cysteine

Interestingly, intracellular cyst(e)ine depletion by the xCT inhibitor erastin or cyst(e)inase administration induced ferroptosis in PDAC, both *in vivo* and *in vitro* (Daher et al., 2019; Badgley et al., 2020). In human PDAC cells, erastin suppressed clonogenicity (Daher et al., 2019), suggesting that, combined with chemotherapy, it bears long-term survival potential, eliminating CSCs.

##### Glutamine

The glutaminase inhibitors 968 and BPTES and, more recently, the orally available CB839 have demonstrated benefits *in vitro* against different PDAC models (Son et al., 2013; Biancur et al., 2017), leading to the design of a phase I clinical trial testing CB839 (Table 1). Unfortunately, glutaminase inhibition seems to be ineffective *in vivo* as a stand-alone treatment, since multiple compensatory mechanisms were activated in response to the inhibitor. However, appropriate dosing of combined treatments could lead to survival benefits as demonstrated *in vitro* (Biancur et al., 2017). Interestingly, combining radiation with BPTES, 968, or the antiashmatic compound Zaprinast, identified as a novel glutaminase inhibitor (Elhammali et al., 2014), diminished PDAC CSCs self-renewal *in vitro* and *in vivo* (Li et al., 2015).

As mentioned earlier, GBMs of the mesenchymal subtype have increased glutamine uptake when compared to non-mesenchymal tumours, which could initiate the use of glutamine metabolic targeting as a therapeutic strategy. Indeed, it was recently shown that CB839 delayed tumour progression in mesenchymal GBM tumour-bearing mice, while having no effect in non-mesenchymal GBM tumour-bearing mice (Oizel et al., 2020), and obtained similar effects when using EGCG, a GDH inhibitor (Oizel et al., 2020). *In vitro* experiments have proven that CB839 selectively impairs the stemness of GSCs expressing high GLS levels (Koch et al., 2020). Since clinical trials using CB839 have resulted in promising advances for triple-negative breast cancer and renal cell carcinoma (Garber, 2016), combining CB839 with standard therapy in GBM could improve patient survival.

In patients with IDH1-mutant GBM cells, glutaminase inhibition with BPTES slows their proliferation, without major effects in wild-type cells (Seltzer et al., 2010). It has been shown that treatment with CB839 radiosensitised IDH1 mutant cells in an *in vivo* model, resulting in tumour growth inhibition of IDH1 mutant gliomas but not of wild-type tumours (McBrayer et al., 2018). These results motivated a clinical trial combining CB839 with radiotherapy and temozolomide in low grade glioma patients with IDH mutations (NCT03528642), but not yet in glioblastoma patients.

In HCC, using CB839 alone did not bear strong effects, even when in highly glutamine addicted HCC cells. However, combining CB839 with a glutamine transporter ASCT2 inhibitor (V-9032), resulted in apoptosis of glutamine-dependent HCC cells *in vitro*, and inhibited tumour growth in HCC xenograft mouse models *in vivo* (Jin et al., 2020)*.* In another pre-clinical study, it was observed that the growth of human CTNNB1-mutated HCC xenografts was delayed by combining Crisantaspase, a drug in clinical use for acute lymphoblastic leukaemia, with the irreversible GS inhibitor MSO (Chiu et al., 2014). These two studies indicate that glutamine depletion may be a pharmacological approach to treat HCC. Moreover, GLS inhibition with BPTES prolonged the survival of a MYC-overexpressing mouse model of liver cancer, with MYC-dependent GLS expression (Xiang et al., 2015).

##### Proline

Another relatively successful dietary intervention in preclinical *in vitro* and *in vivo* works is proline starvation, which was well tolerated by mice. Indeed, prevention and efficacy studies *in vivo* show that a proline-free diet for 1 month significantly reduced tumour initiation and maintenance in cell lines from different tumour types with developed auxotrophy for proline (Sahu et al., 2016).

##### Serine

Serine or glycine dietary restriction did not modify tumour growth in the KPC mouse model (Maddocks et al., 2017), suggesting that PDAC tumours were able to activate SBP to synthesise their own serine. Although new data on human PDAC cells suggested that serine deprivation by dietary intervention, SBP inhibitors or serine uptake may slow tumour growth in patients, intratumoural nerves recruitment into the tumor mass may conflict with this intervention as they could provide serine to the tumour (Banh et al., 2020).

Interestingly, by combining serine restriction and the IDO inhibitor epacadostat in a KPC tumour implanted in immunocompromised mice to discard the well-known immunological effects of IDO inhibition, tumour growth was significantly slowed down, while the single interventions showed no effect (Newman et al., 2021). These results suggest that IDO inhibition, currently tested in numerous clinical trials for PDAC treatment (Table 1), may have anti-tumoural effects by affecting both the immune system and tumour cells.

## Conclusion

Different cancer types show diverse metabolic dependencies and find different ways to adapt to nutrient deprivation, yet, similarities can be observed (Table 2 and Table 3). For instance, both PDAC and GBM CSCs rely mainly on oxidative phosphorylation to produce ATP, but certain subpopulations can switch to glycolysis if needed. The three cancer types discussed here depend on arginine (at least in a subgroup of patients, if not in every patient) for proliferation and motility, rendering them sensitive to arginine deprivation, using ADI-PEG 20. Glutamine is one of the most broadly studied amino acids in several cancers. Different reports have strongly linked glutamine metabolism and maintenance of stemness-related properties via redox control in both PDAC and HCC, which may also be the case for mesenchymal subtypes in GBM. Finally, targeting the enzyme IDO1 seems a very promising strategy in the three cancer types studied for immunosuppression impairing, but most probably such treatment will need to be combined with other chemotherapies currently applied in each cancer type. In addition, IDO1 role and its inhibition effects on stemness and metastasis are unknown, which could also compromise the viability of this approach. Clearly, targeting amino acid metabolism and its transporters in combination with other targeted therapies could bring us a step closer to finding a treatment attacking the most tumourigenic and metastatic subpopulations in several cancer types.

**TABLE 2:** Effects of glucose in the studied cancer types.

GLUCOSE	PDAC	HCC	GBM
CSCs phenotype	OXPHOS Sancho et al. (2015)	Glycolysis & HBP Chen et al. (2013a), Lin et al. (2016)	OXPHOS with plasticity towards glycolysis Vlashi et al. (2011), Shibao et al. (2018)
	Glycolysis related to bad prognosis and metastasis Espiau-Romera et al. (2020); Tian et al., 2020)		
Inhibitors	Metformin inhibits self-renewal Sancho et al. (2015)	Metformin inhibits proliferation and invasion Ferretti et al. (2019)	
	MCT, 2DG inhibit metastasis Roy et al. (2015), Kong et al. (2016)		

**TABLE 3:** Effects of amino acids in the studied cancer types.

		PDAC	HCC	GBM
EAA	BCAAs	↑Proliferation and self-renewal Lee et al. (2019)	↓ Proliferation neovascularisation and stemness Nishitani et al. (2013), Tajiri and Shimizu (2018)	↓ Macrophage activity Silva et al. (2017)
	Methionine		MAT1A ↓ MAT2A↑	MTAP ↓
			↑ HCC development and progression Martínez-Chantar et al. (2002)	↑ Met uptake Palanichamy et al. (2016)
				↑Stemness Zgheib et al. (2019)
	Tryptophan	IDO1 ↑	IDO1 ↑	IDO1 ↑
		↑ Nucleotide synthesis, tumour growth and stemness Newman et al. (2021)	↑ distant metastases and immune suppression Opitz et al. (2020), Shibata et al. (2016)	↑ immune suppression and shorter survival Zhai et al. (2017), (Wainwright et al. (2012), (2014); Lukas et al. (2019)
NEAA/CEAA	Arginine	ARG2 ↑ ASS1 ↑ tumourigenesis, clonogenicity, anchorage-independent cell growth and invasiveness Wang et al. (2016), Zaytouni et al. (2017)	↓ ASS1↑dependency on exogenous Arg for cell proliferation, growth, and survival Ensor et al. (2002)	↓ ASS1 (30% of patients)↑ dependency on exogenous Arg Syed et al. (2013), Mörén et al. (2018) to support cell viability and invasion Pavlyk et al. (2015)
		ADI-PEG 20↓ stemness Kim et al. (2020)	Purified ADI↓ proliferation Takaku et al. (1995)	ADI-PEG 20↑survival and therapeutic synergy with temozolomide in GBM expressing ASS1 Przystal et al. (2018)
	Asparagine	ASNS ↓ Dufour et al. (2012)	ASNS ↓ Zhang et al. (2013)	ASNS ↑
		↑chemoresistance after ASNS upregulation by glucose deprivation, Cui et al. (2007), Pathria et al. (2019)	L-Asparaginase ↓ proliferation ↑ apoptosis. Tardito et al. (2011)	↑ proliferation, invasiveness, resistance to radiation Thomas et al. (2021)
		L-Asparaginase ↓ proliferation *in vitro* and *in vivo* Dufour et al. (2012)		L-Asparaginase ↑ apoptosis ↓ tumour growth Karpel-Massler et al. (2016)
		
		
	Glutamine	CEAA Liang et al. (2016), Halbrook and Lyssiotis, (2017)	CEAA	NEAA, mostly transformed to Glu Tardito et al. (2015)
		ASCT2 ↑ Wang et al. (2019a)	ASCT2 ↑ Sun et al. (2016)	↑ GS for stemness Tardito et al. (2015)
		Gln deprivation ↑ EMT Recouvreux et al. (2020) ↑ apoptosis ↓ self-renewal Li et al. (2015), Liao et al. (2017)	GS ↑ Zhang et al. (2013)↑ Gln induces cell growth Adebayo Michael et al. (2019)↑GLS1 for stemness Li et al. (2019)	
	Proline	↑PRODH1	↑ Pro consumption leading to Hydroxy-Pro accumulation	
		↑ survival, tumourigenicity Olivares et al. (2017)	↑ HCC progression and chemoresistance Tang et al. (2018)	
	Serine	↑PHGDH ↑PSAT1 (60% PDAC cell lines)		↑PHGDH Engel et al. (2020)
		↑ proliferation Banh et al. (2020)		↑proliferation and invasiveness Liu et al. (2013)
